# Oral Microbiota Dominance in Allogeneic Hematopoietic Stem Cell Transplantation: Dynamics, Predictors, and Clinical Implications in a Multicenter Study

**DOI:** 10.3390/microorganisms14102249

**Published:** 2026-10-04

**Authors:** Danielle Amanda Niz Alvarez, Marcus Vinicius Niz Alvarez, Alexandre Soares Ferreira Junior, Welinton Yoshio Hirai, Nathalia Linares Silva, Larissa da Silva Souza Cordeiro, Luiza Dias Machado, Rozana Mesquita Ciconelli, Joao Victor Piccolo Feliciano, Iago Colturato, George Mauricio Navarro Barros, Phillip Scheinberg, Gislane Lelis Vilela de Oliveira

**Affiliations:** 1 Department of Genetics, Microbiology and Immunology, Biosciences Institute (IBB), Sao Paulo State University (UNESP), Botucatu 18618-689, SP, Brazilmarcus.alvarez@unesp.br (M.V.N.A.);; 2 Department of Epidemiology and Biostatistics, Barretos Cancer Hospital, Barretos 14784-400, SP, Brazil; 3 Research Department, Beneficência Portuguesa Hospital (BP), São Paulo 01323-001, SP, Brazil; 4 Bone Marrow Transplant Unit, School of Medicine from Sao Jose do Rio Preto (FUNFARME), Sao Jose do Rio Preto 15090-000, SP, Brazil; 5 Bone Marrow Transplant Unit, Amaral Carvalho Hospital, Jau 17210-070, SP, Brazil; 6 Bone Marrow Transplant Unit, Fundação Pio XII, Barretos Cancer Hospital, Barretos 14780-360, SP, Brazil

**Keywords:** oral microbiome, oral dominance, hematopoietic stem cell transplantation, graft-versus-host disease, dysbiosis

## Abstract

Allogeneic hematopoietic stem cell transplantation (allo-HSCT) is an important therapeutic strategy for hematological malignancies and other diseases but remains associated with substantial morbidity and mortality. Although microbial dominance has been extensively investigated in the gut microbiota, its dynamics and clinical implications in the oral microbiota remain poorly understood. In this prospective multicenter study, longitudinal oral mucosal samples were collected from pre-conditioning to six months post-transplantation in Brazilian patients undergoing allo-HSCT, and microbiota composition was assessed by 16S rRNA gene sequencing. Associations between oral dominance and death within D+180, disease relapse, and acute graft-versus-host disease (aGvHD) were evaluated using competing-risk methods, while predictors of oral dominance were assessed using mixed-effects logistic regression. Among 90 patients, 244 oral samples were analyzed. Alpha diversity significantly decreased during the first 30 days post-allo-HSCT compared with healthy controls. Oral dominance occurred in 87.8% of patients, with *Streptococcus*, *Veillonella*, and *Rothia* being the most frequent dominant genera. *Streptococcus* dominance was associated with a higher cumulative incidence of moderate-to-severe aGvHD, whereas *Rothia* dominance was associated with a higher cumulative incidence of disease relapse. These findings demonstrate substantial oral microbiota disruption during allo-HSCT and suggest that specific taxonomic patterns may be associated with clinically relevant transplant outcomes.

## 1. Introduction

Allogeneic hematopoietic stem cell transplantation (allo-HSCT) is an established therapeutic strategy for several chemo-resistant oncohematological diseases [1,2,3]. Globally, 19,368 allo-HSCT procedures were reported in 2023 across 50 countries and 696 participating centers, reflecting the continuous expansion of transplant activity driven by advances in transplantation protocols and supportive care [4,5]. Despite these advances, allo-HSCT remains associated with significant morbidity and mortality, largely due to complications such as acute graft-versus-host disease (aGvHD), chronic graft-versus-host disease (cGvHD), mucositis, and opportunistic infections [3,6]. Among these complications, aGvHD is one of the main causes of non-relapse mortality following allo-HSCT [7,8].

Given the high morbidity and mortality associated with allo-HSCT, the identification of microbial markers associated with transplant outcomes has emerged as an important field of investigation. In recent years, accumulating evidence has highlighted the role of the microbiota in the allo-HSCT setting, particularly due to its contribution to immune modulation and maintenance of host homeostasis [9,10,11]. The microbiota, defined as the collection of living microorganisms inhabiting a specific ecosystem, actively influences its surrounding microenvironment [12], modulating immune responses and systemic homeostasis [13,14].

The oral cavity constitutes the first interface between mucosal surfaces and the external environment [15] and harbors a highly diverse microbiome composed of more than 700 microbial species, including bacteria, archaea, viruses, fungi, and protozoa. As such, it is considered the second most complex microbial ecosystem in the human body [16,17]. Distinct oral microbiota profiles have been associated with several pathological conditions. In allo-HSCT recipients, bloodstream infections caused by oral microorganisms represent an important source of morbidity and mortality [18]. In addition, oral complications such as mucositis, periodontitis, and gingivitis are frequently observed in these patients and have been associated with infectious outcomes [19,20,21]. Oral dysbiosis, characterized by increased abundance of pathogenic species and disruption of microbial equilibrium, may therefore contribute to both local and systemic complications [22].

Emerging evidence further suggests that oral microbiota characteristics are associated with clinically relevant outcomes following allo-HSCT. Alterations in microbial composition, including an increase in potentially pathogenic bacterial genera such as *Veillonella*, *Streptococcus*, and *Enterococcus*, have been observed throughout the transplantation process and may influence transplant-related outcomes [23,24]. Moreover, reduced oral diversity during the pre-conditioning phase has been associated with an increased risk of relapse [24], while variations in genera such as *Prevotella* spp. and *Actinomyces* spp. have been linked to aGvHD development [25].

Despite the growing interest in the oral microbiota as a potential prognostic marker in allo-HSCT, relatively few studies have specifically investigated the role of bacterial microbial dominance in this context, particularly in relation to aGvHD. Previous microbiota studies defined bacterial dominance as a taxon relative abundance ≥ 30% [24,26,27,28]. However, the optimal threshold for oral microbiota dominance remains to be established across independent cohorts. To date, only a limited number of studies have evaluated bacterial dominance within the oral microbiota of allo-HSCT recipients [23,24,29,30], underscoring the need for further investigation in this still underexplored area. Given the significant mortality associated with allo-HSCT, elucidating the relationship between microbial dominance events and transplant-related outcomes may contribute to the development of improved prognostic and risk-stratification strategies [18,23].

In our previous study, we investigated the prognostic significance of intestinal dominance events in the allo-HSCT setting [28]. Building upon these findings and considering the limited literature addressing the oral microbiota in this context, the present study aimed to characterize bacterial dominance events within the oral microbiota of patients undergoing allo-HSCT, evaluate their association with clinical outcomes, and identify clinical predictors associated with the occurrence of these events.

## 2. Patients, Materials and Methods

### 2.1. Study Design, Ethics, and Sample Collection

This prospective, observational, multicenter cohort study included patients undergoing allo-HSCT. The study was approved by the Research Ethics Committee of Sao Paulo State University (process number 5.138.190/2021) and conducted in accordance with the Declaration of Helsinki. Written informed consent was obtained from all adult participants prior to enrollment. For participants under 18 years of age, informed assent was obtained from the patient and written informed consent was provided by their legal representative.

Patients aged ≥12 years undergoing allo-HSCT who provided at least one oral sample during treatment and had clinical data available in REDCap were eligible for inclusion. Exclusion criteria: (1) Cord blood transplantation, (2) absence of oral sample collection during the 6-month follow-up period; and (3) withdrawal from the study. Additionally, 16 healthy individuals without comorbidities and not receiving antibiotic therapy were recruited as controls.

Oral mucosal samples were collected longitudinally at seven predefined time points: before the conditioning regimen (D−7), on the day of stem cell infusion (D0), and at D+30, D+60, D+90, and D+180 post-transplantation, as well as at the time of aGvHD diagnosis. Samples were obtained by swabbing the buccal mucosa and the dorsal surface of the tongue with a single swab for 10 s in each region [31,32,33], resulting in a composite sample representing both sites. After collection, samples were stored at −80 °C until DNA extraction.

The aGvHD was graded according to the MAGIC criteria, which take into account the affected organs, the severity of organ involvement, and the overall clinical impact of symptoms, particularly regarding the need for therapeutic interventions [34].

### 2.2. Oral Microbiota 16S Sequencing and Bioinformatics Pipeline

Oral microbial DNA was extracted using the QIAamp Fast DNA Stool Mini Kit (Qiagen, Redwood, CA, USA), following the manufacturer’s instructions with minor adaptations. The same standardized DNA extraction and PCR protocols were applied to all samples to minimize technical variability. DNA concentration was quantified using the Qubit dsDNA HS Assay Kit (Thermofisher, Waltham, MA, USA). A minimum DNA concentration of 1 ng/mL was required for samples to proceed to sequencing. Among the microorganisms present in the oral microbiota, this study specifically targeted the bacterial component. The V3–V4 regions of the bacterial 16S rRNA gene were amplified using a two-step PCR protocol with the following primers: 341F: 5′-TCGTCGGCAGCGTCAGATGTGTATAAGAGACAGCCTACGGGNGGCWGCAG; 805R: 5′-GTCTCGTGGGCTCGGAGATGTGTATAAGAGACAGGACTACHVGGGTATCTAATCC. PCR amplification was performed using 2X Platinum SuperFi II PCR Master Mix (Thermofisher) under the following cycling conditions: initial denaturation at 98 °C for 30 s, followed by 30 cycles of 98 °C for 10 s, 55 °C for 10 s, and 72 °C for 30 s. PCR negative controls were included to monitor potential contamination during amplification. Sequencing was performed on the Illumina MiSeq platform (Illumina, San Diego, CA, USA).

Sequencing quality was initially assessed using FastQC v0.11.9 [35], including evaluation of adapter content, average read quality, and nucleotide composition. Reads were processed with Trimmomatic v0.39 [36] according to the quality metrics obtained, including paired-end read handling, trimming of the first 20 bases, exclusion of reads with mean quality scores below Q30, and removal of reads shorter than 100 bp. A minimum sequencing depth of 100,000 reads per sample was required for inclusion in downstream analyses. Paired-end reads were merged using FLASH v1.2.11 [37] with a minimum overlap of 50 bp. Processed sequences were imported into QIIME2 v2024.2 [38]. Sequence denoising and amplicon sequence variant (ASV) inference were performed using DADA2. No additional ASV filtering, contaminant removal, batch-effect correction, or low-biomass filtering was applied. Taxonomic assignment was conducted using the Human Oral Microbiome Database [39] with the classify-sklearn method. Phylogenetic reconstruction was performed using the align-to-tree-mafft-fasttree pipeline.

Alpha diversity indices were normalized using a Box–Cox transformation, with the transformation parameter (λ) estimated by maximum likelihood using the car package for R. Subsequently, a linear mixed-effects model was fitted using the lmer function from the lme4 package for R, with collection time points as a fixed effect and patient as a random effect. Post hoc pairwise comparisons were performed using estimated marginal means and *p*-values were adjusted using the Benjamini–Hochberg procedure with the emmeans package for R. Beta diversity was assessed by Principal Coordinates Analysis (PCoA, using ape package for R) based on weighted and unweighted UniFrac distance matrices, followed by post hoc pairwise PERMANOVA test using the self-named R package. Statistical analyses were conducted in R v4.4.1 using RStudio Serverv2023.12 (Ocean Storm) [40,41]. Data processing and visualization were performed using the tidyverse v2.0.0 package [42].

The sequencing data generated in this study have been deposited in the NCBI BioProject database under accession number PRJNA1467748. The data are currently under restricted access and will be made publicly available upon publication of this manuscript.

### 2.3. Statistical Analysis

Descriptive analyses were stratified according to oral dominance status. Oral dominance was defined as a relative abundance of ≥30% for any bacterial genus, consistent with previous intestinal microbiota studies for comparison [26,27,28]. The patient-level distribution of oral dominance is shown in Supplementary Figure S3. Associations between oral dominance and clinical variables were evaluated using the chi-square or Fisher’s exact test for categorical variables and the Wilcoxon rank-sum test for continuous variables. An additional analysis evaluating the association between oral dominance by *Streptococcus* and positive blood culture results (*Streptococcus* vs. other bacterial genera) was performed using the chi-square or Fisher’s exact test (see Supplementary Table S3).

Competing-risks analyses were performed to assess the association between oral dominance by *Streptococcus*, *Veillonella*, *Rothia*, or any bacterial genus and the cumulative incidence of death within D+180, moderate-to-severe aGvHD (grades II–IV), and disease relapse. For these analyses, dominance was considered an ever-dominant exposure, defined as the occurrence of at least one sample meeting the predefined dominance criterion during follow-up. The three clinical outcomes were considered mutually competing events, and patients were classified according to the first event occurring chronologically during follow-up. Thus, the occurrence of one outcome precluded subsequent classification as another competing event. Cumulative incidence functions were estimated accounting for the competing occurrence of the three outcomes and compared between dominance groups using Gray’s test.

Potential demographic and clinical predictors of oral dominance were evaluated using univariate and multivariate mixed-effects logistic regression models, with patient included as a random effect to account for repeated oral samples collected from the same individual. Due to missing data, the number of oral samples included in each univariate analysis varied according to the availability of data for each predictor. For the multivariate model, which included all demographic and clinical variables evaluated in the univariate analyses, only oral samples from patients with complete data for all included variables were analyzed, resulting in a total of 221 oral samples. All analyses were performed using R in RStudio, and *p*-values < 0.05 were considered statistically significant.

## 3. Results

### 3.1. Patients’ Clinical and Demographic Characteristics

A total of 244 oral samples were collected from 90 patients undergoing allo-HSCT. The distribution of samples according to transplantation time point and participating center is summarized in Supplementary Table S1 and Figure S4. Demographic and clinical characteristics stratified by oral dominance status are presented in Table 1, while data from healthy controls are shown in Supplementary Table S2. No significant differences in demographic characteristics were observed between groups. Clinically relevant outcomes observed during the study period included aGvHD in 21/90 patients (23.3%), disease relapse in 9/90 patients (10%), and mortality within D+180 in 25/90 patients (27.8%).

### 3.2. Alpha and Beta Diversity and Prevalence of Oral Dominance

**Figure 1:**
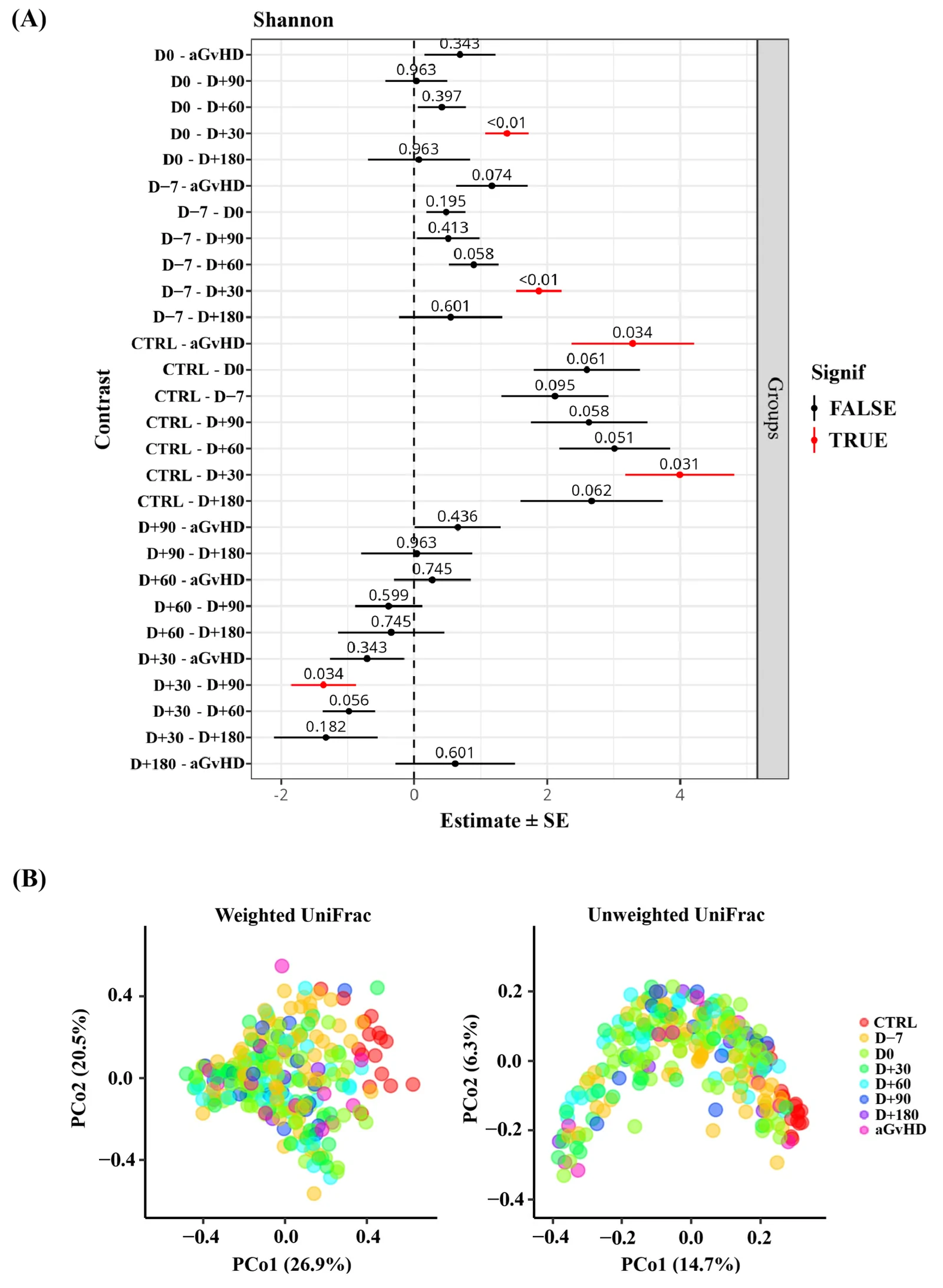
Alpha and beta-diversity parameters in allo-HSCT. (**A**) Shannon index across different time points. (**B**) Principal coordinates analysis (PCoA) based on weighted and unweighted UniFrac distance matrices. aGvHD = acute graft-versus-host disease; CTRL = control; D = day.

Statistically significant differences in alpha diversity were observed throughout the allo-HSCT course and when compared with the control group (CTRL). Lower diversity was observed at D+30 and aGvHD when compared to CTRL (*p* = 0.031 and *p* = 0.034, respectively; see Figure 1A). During the allo-HSCT course, D+30 showed lower diversity when compared to the baseline D−7, D0 and D+90 (*p* < 0.01, *p* < 0.01 and *p* = 0.034, respectively; see Figure 1A). Regarding beta diversity, no statistically significant differences were observed in the composition across the different transplant time points. However, oral bacterial composition differed between healthy individuals and allo-HSCT patients (PERMANOVA, *p*-Values = 0.036; see Figure 1B). Pairwise post hoc comparisons are presented in Supplementary Tables S4 and S5.

**Figure 2:**
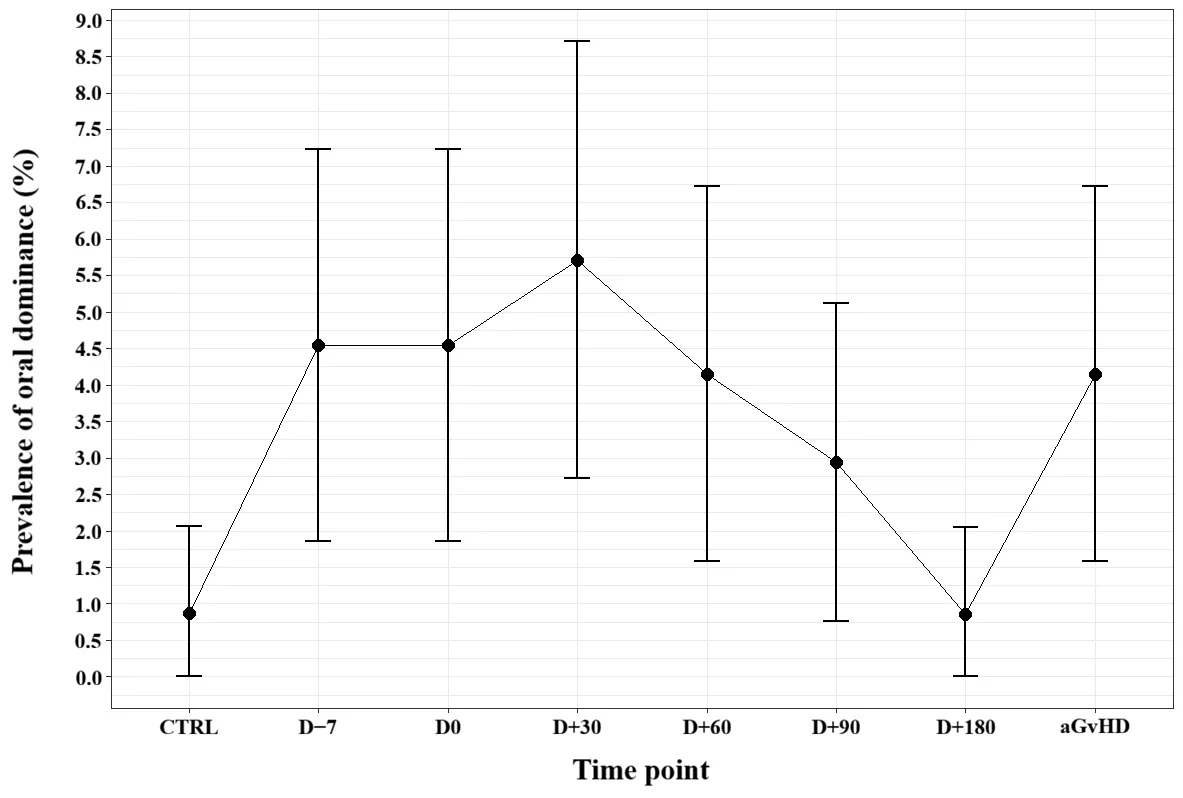
Prevalence of oral dominance by any bacterial genus among oral samples (n = 244) collected at different time points. Error bars represent 95% confidence intervals calculated using the Wilson score method for proportions, which is appropriate for reduced sample sizes at later time points. aGvHD = acute graft-versus-host disease; CTRL = control; D = day.

**Figure 3:**
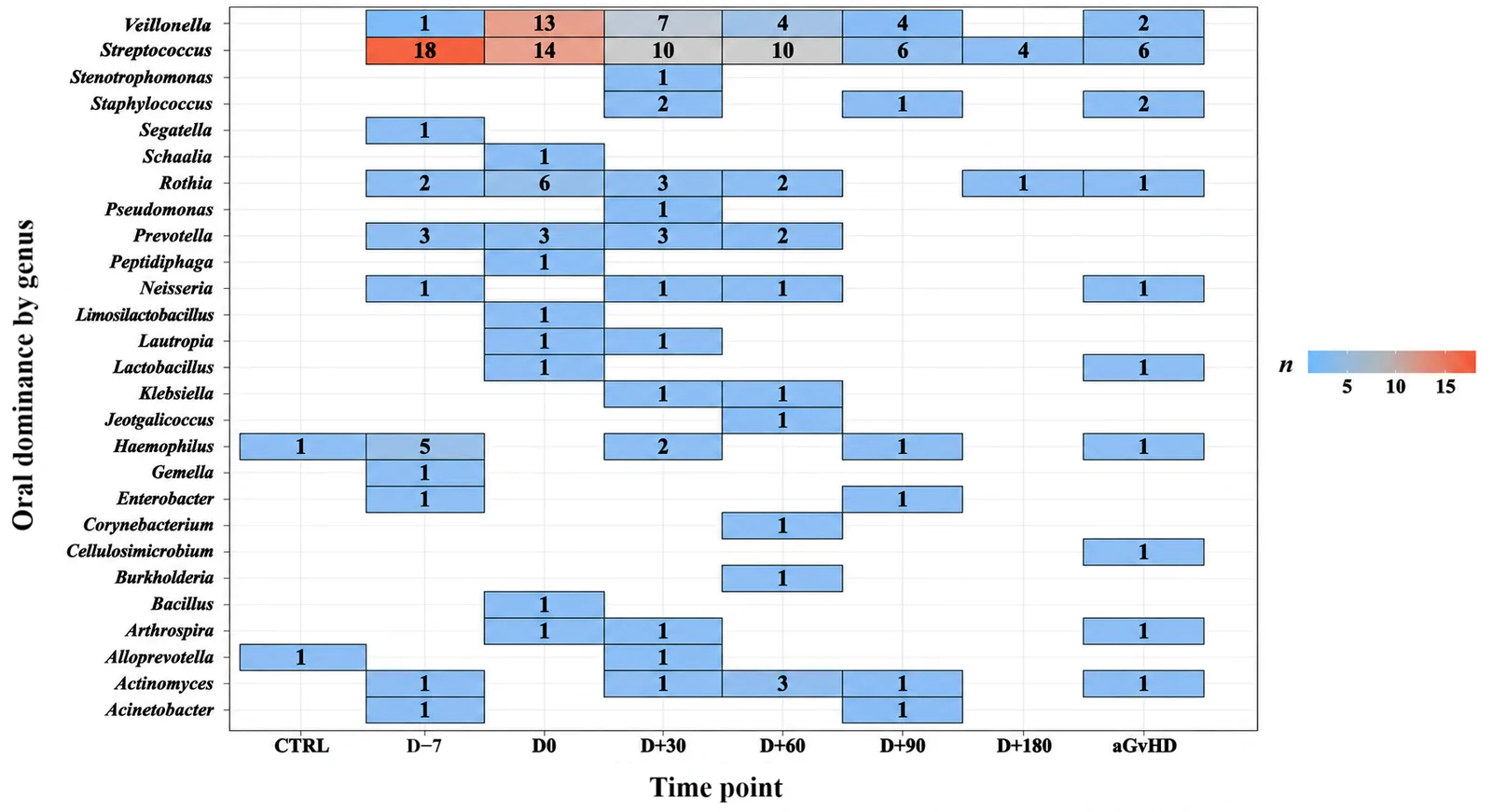
Oral dominance by specific bacterial genus across different time points based on sample counts. aGvHD = acute graft-versus-host disease; CTRL = control; D = day.

The sample-based prevalence of oral dominance by any bacterial genus is shown in Figure 2, whereas oral dominance by specific bacterial genera based on sample counts is presented in Figure 3, stratified across different time points. Oral dominance was already evident prior to transplantation (D−7) and at the time of stem cell infusion (D0), with prevalence increasing compared to controls. The highest prevalence was observed at D+30, followed by a progressive decline at later time points. Among the 27 identified genera (see Supplementary Tables S6 and S7), three accounted for the highest number of dominant samples: *Streptococcus* (*n* = 68), *Veillonella* (*n* = 31), and *Rothia* (*n* = 15).

### 3.3. Associations Between Oral Dominance and Clinical Outcomes

We evaluated the associations between oral dominance by the three main bacterial genera and clinical outcomes, including death within D+180, moderate-to-severe aGvHD (grades II–IV), and disease relapse.

**Figure 4:**
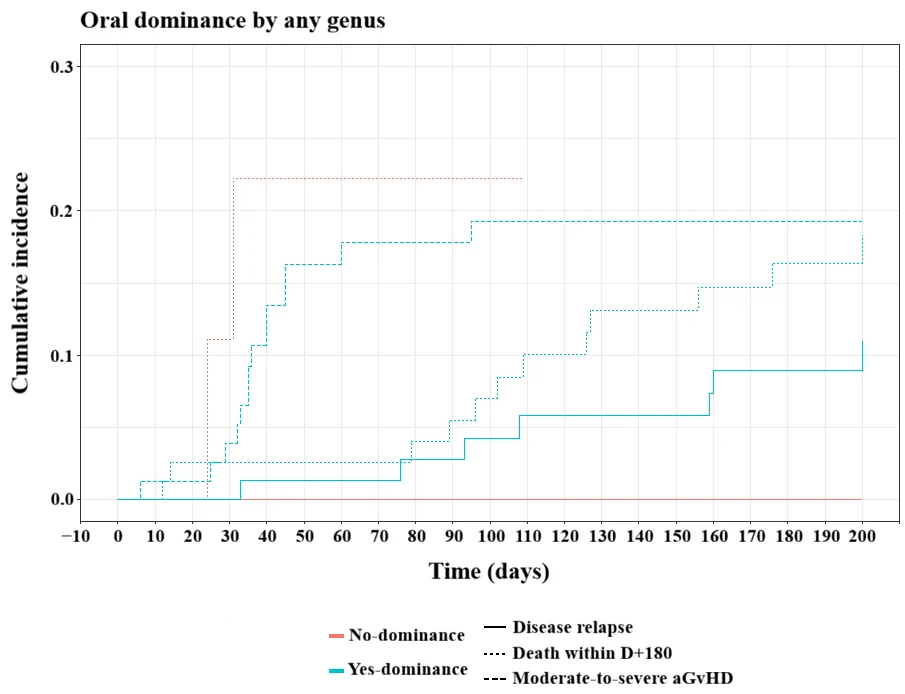
Cumulative incidence of clinical outcomes according to oral dominance by any genus. aGvHD = acute graft-versus-host disease; D = day.

**Figure 5:**
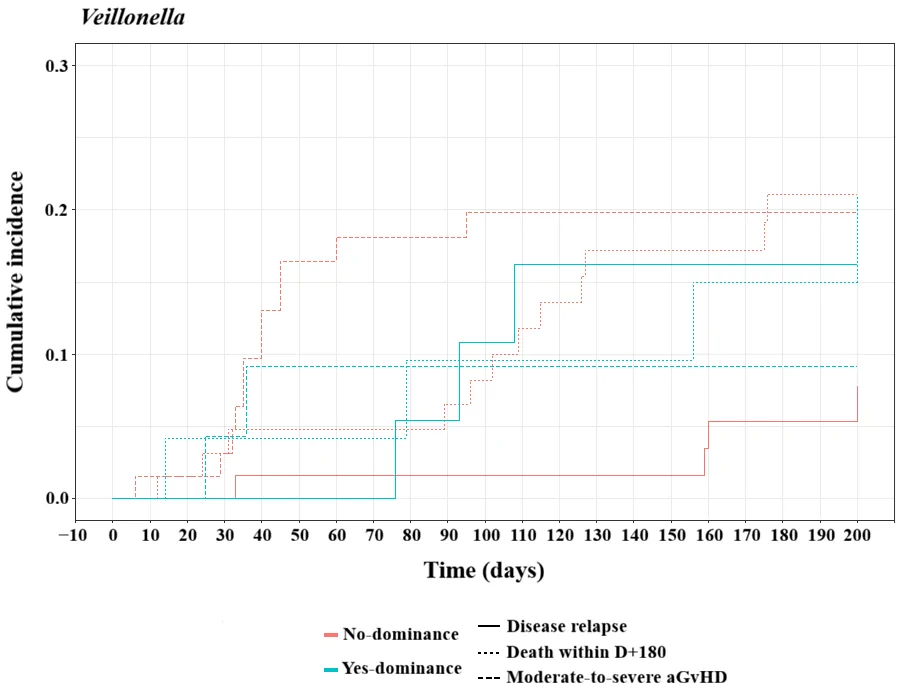
Cumulative incidence of clinical outcomes according to *Veillonella* oral dominance. aGvHD = acute graft-versus-host disease; D = day.

When patients were stratified according to overall oral dominance status, no statistically significant associations were observed with any clinical outcome (all *p* > 0.06; see Figure 4). Similar findings were obtained when oral dominance by specific genera was evaluated, with no statistically significant associations for *Veillonella* (all *p* > 0.2; see Figure 5).

**Figure 6:**
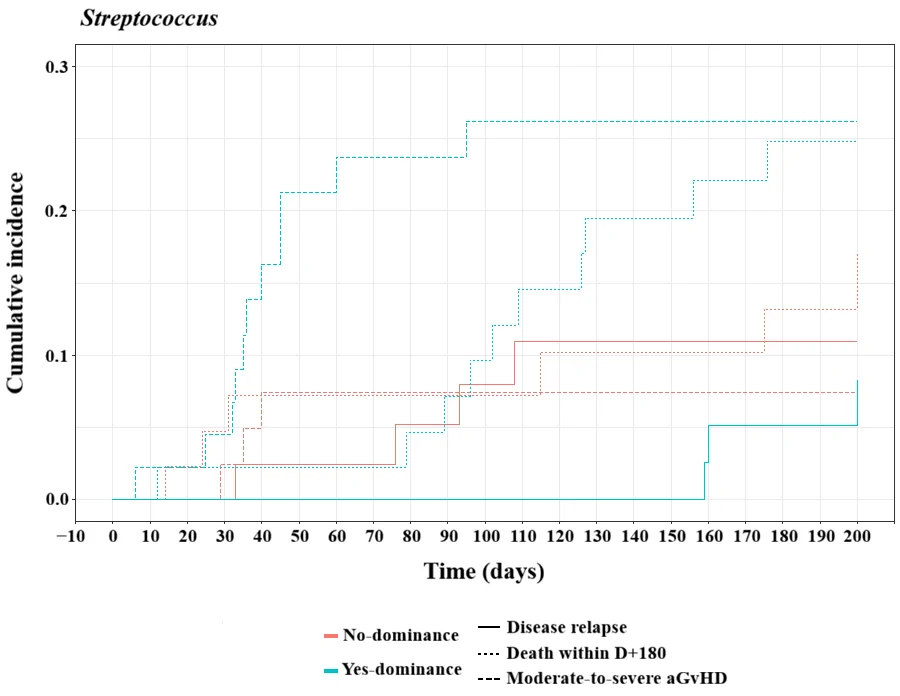
Cumulative incidence of clinical outcomes according to *Streptococcus* oral dominance. aGvHD = acute graft-versus-host disease; D = day.

**Figure 7:**
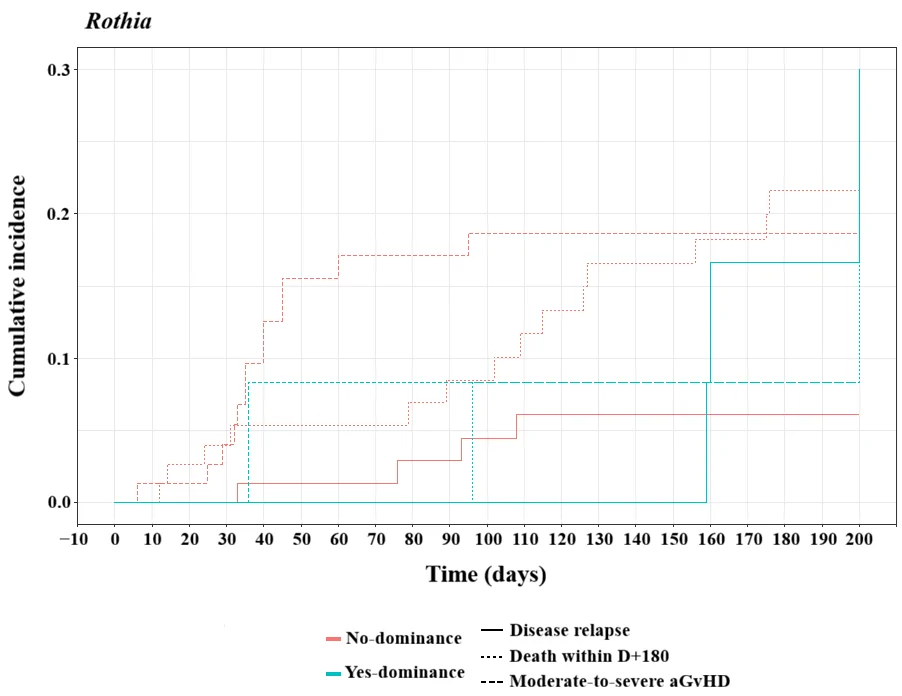
Cumulative incidence of clinical outcomes according to *Rothia* oral dominance. aGvHD = acute graft-versus-host disease; D = day.

In contrast, for *Streptococcus* (see Figure 6), although no statistically significant associations were observed with death within D+180 (*p* = 0.4) or disease relapse (*p* = 0.52), dominance by this genus was associated with a higher cumulative incidence of moderate-to-severe aGvHD (*p* = 0.029). For *Rothia* (see Figure 7), although no statistically significant associations were observed with death within D+180 (*p* = 0.69) or moderate-to-severe aGvHD (*p* = 0.39), dominance by this genus was associated with a higher cumulative incidence of disease relapse (*p* = 0.046).

All comparisons between oral dominance status, whether by any genus or by a specific genus, and clinical outcomes are presented in Supplementary Material (Supplementary Tables S8–S11).

### 3.4. Predictors of Oral Microbiota Dominance

Univariate and multivariate mixed-effects logistic regression analyses identifying predictors of oral dominance by any bacterial genus are presented in Table 2 and Table 3, respectively. In univariate analysis, smoking status was associated with oral dominance, with former smokers showing lower odds compared to never smokers (OR = 0.81; 95% CI, 0.66–1.00; *p* = 0.045; see Table 2). In multivariate analysis (see Table 3), the association of smoking status did not remain significant after adjustment for other clinical covariates. Additionally, no other significant associations were identified.

## 4. Discussion

Considering the clinical relevance of transplant-related complications in allo-HSCT recipients, understanding oral bacterial microbiota dynamics may contribute to the identification of microbiota markers associated with clinically relevant outcomes. In our cohort, we observed a marked reduction in oral bacterial diversity during transplantation and a high patient-level prevalence of oral dominance, with 87.8% of patients (*n* = 79) presenting at least one dominance event throughout the transplantation course, suggesting that oral dysbiosis is a frequent feature in the allo-HSCT setting. We further identified that *Streptococcus* dominance was associated with a higher cumulative incidence of moderate-to-severe aGvHD, whereas *Rothia* dominance was associated with a higher cumulative incidence of disease relapse. However, dominance by *Veillonella* was not independently associated with major transplant outcomes.

The decrease in alpha diversity observed during the early transplantation period is consistent with previous studies demonstrating profound oral bacterial microbiota disruption following conditioning and stem cell infusion [18,23,24,25,30,33,43,44,45]. Collectively, these findings support the concept that allo-HSCT induces a substantial ecological disturbance in the oral cavity, likely driven by conditioning-related mucosal injury, immunosuppression, antimicrobial exposure, and impaired immune reconstitution. In addition to reduced diversity, we observed differences in bacterial composition between healthy controls and allo-HSCT recipients, further reinforcing the impact of transplantation and immunosuppression on oral microbial ecology. However, these findings may also be influenced by the differences in age and smoking history between the groups, as discussed later in this section.

Within this dysbiotic context, oral dominance emerged as a highly prevalent feature in our cohort. The biological idea of bacterial dominance was first described in a study investigating antibiotic-induced intestinal dominance by a single bacterial taxon that may precede bloodstream infection in allo-HSCT patients [46]. However, the definition of bacterial dominance was only introduced a few years later in studies of the gut microbiota in allo-HSCT recipients using short-amplicon 16S rRNA sequencing, where dominance was defined as the presence of a single bacterial genus accounting for at least 30% of the total relative abundance and used as a proposed threshold [26]. Although this threshold is inherently arbitrary, it has become widely adopted in transplantation microbiome research because it provides a standardized framework for identifying ecological disruption characterized by the dominance of a single taxon. In the present study, we applied the same definition to the oral bacterial microbiota to facilitate comparison with previous transplantation studies while acknowledging that the optimal threshold for oral dominance remains to be established.

While bacterial dominance has been extensively investigated in the gut microbiota of allo-HSCT recipients, particularly in association with acute and chronic GvHD, bacteremia, and mortality [26,27,28,47], its role in the oral microbiota remains comparatively underexplored. Nevertheless, the available literature consistently suggests that oral dominance events are frequent during transplantation. A recent systematic review from our group demonstrated that only a minority of studies evaluating oral bacterial microbiota dynamics specifically investigated dominance events, despite prevalence rates ranging from 59% to 100% across cohorts [10]. Similarly, previous longitudinal studies reported that most allo-HSCT recipients experienced at least one oral dominance event during transplantation [24]. Together, these findings suggest that oral dominance may represent a common manifestation of oral dysbiosis during allo-HSCT, although methodological heterogeneity between studies may influence reported prevalence rates (study design, sampling sites, allo-HSCT time points).

The dominant genera identified in our cohort include *Streptococcus*, *Veillonella*, and *Rothia.* Additionally, previous studies have identified *Neisseria*, *Staphylococcus*, *Lactobacillus*, and *Enterococcus* among the most frequently dominant genera in allo-HSCT recipients [10,24,30]. Notably, genera such as *Streptococcus*, *Veillonella*, *Rothia*, and *Neisseria* are common commensals of the healthy oral microbiota but may undergo ecological expansion under dysbiotic conditions associated with mucosal injury, antimicrobial exposure, and immune dysfunction. A recent study proposed that the increased detection of oral *Streptococcus* in fecal samples of allo-HSCT recipients primarily reflects intestinal microbiota depletion rather than true intestinal colonization by this genus, suggesting that its relative increase may represent a marker of dysbiosis and ecosystem disruption [48]. Although our study evaluated only the oral bacterial microbiota, the high frequency of *Streptococcus* dominance observed in our cohort is consistent with this concept and further supports the potential role of this genus as an indicator of microbial imbalance. In contrast, *Staphylococcus* and particularly *Enterococcus* are more commonly associated with dysbiosis and opportunistic infections in immunocompromised hosts. The variations in dominant taxa across studies likely reflect differences in sampling sites, geographic populations, conditioning regimens, antimicrobial use, and baseline bacterial microbiota composition [11,23,24,25]. Such variability highlights the complexity of defining universal microbial patterns in allo-HSCT recipients.

Regarding the predominance of *Streptococcus* in our cohort, we evaluated whether oral *Streptococcus* dominance was associated with positive blood cultures for *Streptococcus* or other bacterial genera (Supplementary Table S3). One study demonstrated that intestinal dominance by a bacterial genus following antibiotic exposure was associated with an increased risk of subsequent bacteremia caused by the same dominant genus, highlighting the clinical relevance of microbial dominance in allo-HSCT recipients [26]. Although the association between oral *Streptococcus* dominance and *Streptococcus* bloodstream infection did not reach statistical significance, 4 of the 5 *Streptococcus* bloodstream infection episodes occurred among patients with oral *Streptococcus* dominance, whereas bloodstream infections caused by other bacterial genera were more evenly distributed between patients with and without *Streptococcus* dominance. This observed trend may warrant further investigation in larger prospective studies.

While oral bacterial dominance may have prognostic relevance in allo-HSCT, dominance by *Veillonella* was not associated with transplant outcomes in our cohort. However, *Streptococcus* and *Rothia* dominance status were associated with moderate-to-severe aGvHD and disease relapse, respectively. To our knowledge, no previous studies have specifically associated *Rothia* dominance with disease relapse in allo-HSCT recipients. These findings are consistent with previous studies reporting that oral dominance by any genus is associated with an increased risk of relapse compared with patients without oral dominance [24,29]. In those studies, *Rothia* was identified among the dominant genera but was not specifically associated with clinical outcomes. The association between *Streptococcus* dominance and moderate-to-severe aGvHD observed in our cohort is consistent with previous reports linking increased *Streptococcus* abundance to aGvHD [30]. Other studies have also linked oral microbiota alterations to lower survival, increased relapse risk, and mucositis, particularly involving an increase in *Enterococcus faecalis* abundance, whereas higher *Veillonella* abundance has been associated with lower aGvHD risk [24,30,49].

Taken together, our findings suggest that the prognostic significance of oral dominance may vary substantially between cohorts and may depend not only on the dominant taxa involved, but also on broader ecological and clinical factors, including antimicrobial exposure, host immune status, and geographic or population-specific characteristics. In this context, dominance alone may not fully capture the complexity of oral microbiota alterations associated with transplantation outcomes. Rather, microbial interactions, ecological resilience, and community-wide compositional changes may represent more informative biomarkers of dysbiosis-related complications.

Given the high prevalence of oral dominance in our cohort, we investigated factors associated with its occurrence and identified smoking status as a significant predictor in univariate mixed-effects logistic regression. Previous studies have shown that smoking induces persistent alterations in oral bacterial composition [50,51]. However, this association was no longer significant after multivariable adjustment, suggesting that the observed effect may have been confounded by other clinical variables.

Our study is strengthened by its multicenter design and longitudinal evaluation of oral bacterial microbiota dynamics throughout different stages of allo-HSCT in a relatively large cohort. In addition, it expands the limited literature on oral dominance occurrence in allo-HSCT recipients.

Nevertheless, some limitations should be acknowledged. Methodological heterogeneity across studies, particularly regarding the lack of consensus on the optimal oral sampling site [18,24,25,30,31,43], may limit comparisons between cohorts. Different oral niches harbor distinct microbial communities [17,52], and sampling strategies therefore influence the observed microbial composition. However, this approach may have obscured site-specific microbial signals. The younger age and absence of smoking among healthy controls should also be considered when interpreting comparisons with allo-HSCT recipients. This profile reflects the strict criteria adopted to define a healthy control group, as aging is associated with physiological changes in the oral mucosa, increased susceptibility to oral conditions, and changes in the oral microbiome, as well as a higher prevalence of systemic conditions and medication use that may influence microbial composition [53,54]. Similarly, smoking was not considered compatible with the criteria for a healthy control group because of its known effects on the oral microbiota and its potential to compromise oral mucosal integrity and alter the oral microbial environment [50,51].

Other limitations include the unequal distribution of samples across time points and incomplete clinical data, particularly regarding bloodstream infections and post-transplant medication exposure, including immunosuppressive and antibiotic use. These factors may influence the oral microbial ecosystem and could not be comprehensively incorporated into the present analyses because complete longitudinal data were not available for all patients. Future studies incorporating systematically collected medication exposure and immune-related parameters will be important to further characterize the determinants and clinical implications of oral microbiota changes following allo-HSCT. The absence of integrated multi-omics analyses may also have limited a more comprehensive assessment of the clinical and functional implications of oral dominance.

## 5. Conclusions

Overall, our findings demonstrate that oral bacterial dominance is a frequent feature of oral dysbiosis during allo-HSCT, particularly involving genera such as *Streptococcus*, *Veillonella*, and *Rothia*. Although only *Streptococcus* and *Rothia* dominance showed associations with specific transplant outcomes in our cohort, the study reinforces the complexity of oral bacterial microbiota dynamics during transplantation and suggests that broader ecological characteristics may be more relevant than dominance alone in shaping clinical outcomes. Future multicenter studies integrating longitudinal microbiome profiling, functional analyses, and detailed clinical metadata will be essential to clarify the prognostic relevance of oral dysbiosis and to identify robust bacterial microbiota-based biomarkers in allo-HSCT recipients.

## Figures and Tables

**Table 1:** Demographic and clinical characteristics stratified by oral dominance status.

Variable	No-Dominance (*n* = 11)	Oral Dominance (*n* = 79)	Total (*N* = 90)	*p*-Value
Sex				0.5
Female	4 (36%)	37 (47%)	41 (46%)	
Male	7 (64%)	42 (53%)	49 (54%)	
Age (years)				0.2
Mean (SD)	44 (16)	38 (15)	39 (15)	
Median	48	37	38	
Range	13–60	12–73	12–73	
Body mass index				0.2
Mean (SD)	28.3 (4.5)	26.3 (5.8)	26.5 (5.6)	
Median	28.1	25.5	25.8	
Range	18.8–36.0	15.0-40.7	15.0-40.7	
Ethnicity				>0.9
Caucasian	7 (64%)	48 (61%)	55 (61%)	
Afrodescendant	4 (36%)	31 (39%)	35 (39%)	
Smoking status				0.05
Never Smokers	6 (55%)	62 (78%)	68 (76%)	
Current Smokers	1 (9.1%)	5 (6.3%)	6 (6.7%)	
Former Smokers	4 (36%)	6 (7.6%)	10 (11%)	
Hospitals				0.8
1-FUNFARME *	5 (45%)	25 (32%)	30 (33%)	
2-FACJ *	2 (18%)	19 (24%)	21 (23%)	
3-HCB *	0 (0%)	8 (10%)	8 (8.9%)	
4-BP *	4 (36%)	27 (34%)	31 (34%)	
Baseline disease				0.5
Acute Myeloid Leukemia	5 (45%)	33 (42%)	38 (42%)	
Acute Lymphoid Leukemia	1 (9.1%)	18 (23%)	19 (21%)	
Chronic Myeloid Leukemia	1 (9.1%)	3 (3.8%)	4 (4.4%)	
Chronic Lymphoid Leukemia	0 (0%)	1 (1.3%)	1 (1.1%)	
Hodgkin’s Lymphoma	1 (9.1%)	1 (1.3%)	2 (2.2%)	
Non-Hodgkin’s Lymphoma	0 (0%)	1 (1.3%)	1 (1.1%)	
Aplastic Anemia	2 (18%)	7 (8.9%)	9 (10%)	
Sickle Cell Anemia	0 (0%)	5 (6.3%)	5 (5.6%)	
Others	1 (9.1%)	10 (13%)	11 (12%)	
Donor type				0.3
Matched Related	1 (9.1%)	28 (35%)	29 (32%)	
Matched Unrelated	1 (9.1%)	6 (7.6%)	7 (7.8%)	
Mismatched Related	1 (9.1%)	3 (3.8%)	4 (4.4%)	
Haploidentical	8 (73%)	40 (51%)	48 (53%)	
Mismatched Unrelated	0 (0%)	2 (2.5%)	2 (2.2%)	
Conditioning regimen				0.083
Myeloablative	1 (9.1%)	30 (38%)	31 (34%)	
Reduced Intensity	8 (73%)	32 (41%)	40 (44%)	
Nonmyeloablative	2 (18%)	17 (22%)	19 (21%)	
Stem Cell Source				0.3
Peripheral Blood	10 (91%)	57 (72%)	67 (74%)	
Bone Marrow	1 (9.1%)	22 (28%)	23 (26%)	

* FUNFARME: Foundation of the Regional Medical School of Sao Jose do Rio Preto; FACJ: Amaral Carvalho Foundation; HCB: Pio XII Foundation-Barretos Cancer Hospital; BP: Beneficencia Portuguesa Hospital/São Paulo. SD = Standard deviation.

**Table 2:** Univariate Mixed-Effects Logistic Regression Analysis of Predictors of Oral Dominance.

Variable	*n*	OR	95% CI	*p*-Value
Sex	242			
Female	-	-	-	-
Male	-	1.01	0.90–1.14	0.8
Age (years)	241	1.00	0.99–1.00	0.2
Body mass index	242	1.01	1.0–1.02	0.3
Smoking status	222			
Never Smokers	-	-	-	-
Current Smokers	-	0.97	0.72–1.30	0.8
Former Smokers	-	0.81	0.66–1.00	0.045
Baseline disease	242			
Leukemias	-	-	-	-
Others	-	1.00	0.87–1.15	>0.9
Conditioning regimen	242			
Myeloablative	-	-	-	-
Reduced Intensity	-	0.88	0.77–1.01	0.063
Nonmyeloablative	-	0.89	0.75–1.06	0.2
Stem Cell Source	242			
Peripheral Blood	-	-	-	-
Bone Marrow	-	1.01	0.88–1.17	0.9

OR = Odds ratio; CI = Confidence interval; *n* = number of oral samples.

**Table 3:** Multivariate Mixed-Effects Logistic Regression Analysis of Predictors of Oral Dominance.

Variable	*n*	OR	95% CI	*p*-Value
Sex	221			
Female	-	-	-	-
Male	-	1.01	0.56–1.80	>0.9
Age (years)	221	0.98	0.96–1.01	0.2
Body mass index	221	1.04	0.98–1.11	0.2
Smoking status	221			
Never Smokers	-	-	-	-
Current Smokers	-	1.42	0.35–5.81	0.6
Former Smokers	-	0.42	0.17–1.06	0.065
Baseline disease	221			
Leukemias	-	-	-	-
Others		0.89	0.40–1.97	0.8
Conditioning regimen	221			
Myeloablative	-	-	-	-
Reduced Intensity	-	0.60	0.29–1.25	0.2
Nonmyeloablative	-	0.82	0.31–2.19	0.8
Stem Cell Source	221			
Peripheral Blood	-	-	-	-
Bone Marrow	-	1.02	0.47–2.18	>0.9

OR = Odds ratio; CI = Confidence interval; *n* = number of oral samples.

## Data Availability

The original data presented in the study are openly available in the NCBI BioProject database at accession number PRJNA1467748.

## Supplementary Materials

The following supporting information can be downloaded at: https://www.mdpi.com/article/10.3390/microorganisms14102249/s1, Supplementary Figure S1. Read counts per sample across the preprocessing workflow (Input, Trimmomatic, and FLASH); Supplementary Figure S2. Read counts per sample across the DADA2 denoising workflow (Input, Filtered, Denoised, and Non-chimeric); Supplementary Figure S3. Patient-level distribution of oral dominance events during allo-HSCT; Supplementary Figure S4. Patient-level distribution of oral samples during allo-HSCT course; Supplementary Table S1. Distribution of oral samples across different time points and by medical center; Supplementary Table S2. Demographic characteristics of the control group; Supplementary Table S3. Association between oral *Streptococcus* dominance and blood culture results; Table S4. Weighted Unifrac pairwise post hoc comparisons; Table S5. Unweighted Unifrac pairwise post hoc comparisons; Supplementary Table S6. Distribution of dominant bacterial genera in allo-HSCT patients and healthy controls; Supplementary Table S7. Distribution of dominant bacterial genera across different time points during the allo-HSCT course; Supplementary Table S8. Cumulative incidence estimates of clinical outcomes according to oral dominance status during follow-up; Supplementary Table S9. Cumulative incidence estimates of clinical outcomes according to *Streptococcus* oral dominance status during follow-up; Supplementary Table S10. Cumulative incidence estimates of clinical outcomes according to *Veillonella* oral dominance status during follow-up; Supplementary Table S11. Cumulative incidence estimates of clinical outcomes according to *Rothia* oral dominance status during follow-up.
