# Supplementary material for: Oral Microbiota Dominance in Allogeneic Hematopoietic Stem Cell Transplantation: Dynamics, Predictors, and Clinical Implications in a Multicenter Study
Source: Microorganisms. 2026 Oct 4;14(10):2249. doi: 10.3390/microorganisms14102249 (PMC13707435; doi:10.3390/microorganisms14102249)
Supplement: Supplementary file 1 [file microorganisms-14-02249-s001.zip › microorganisms-4561605-supplementary.pdf]

Supplementary material

1. Sequencing and Processing Data

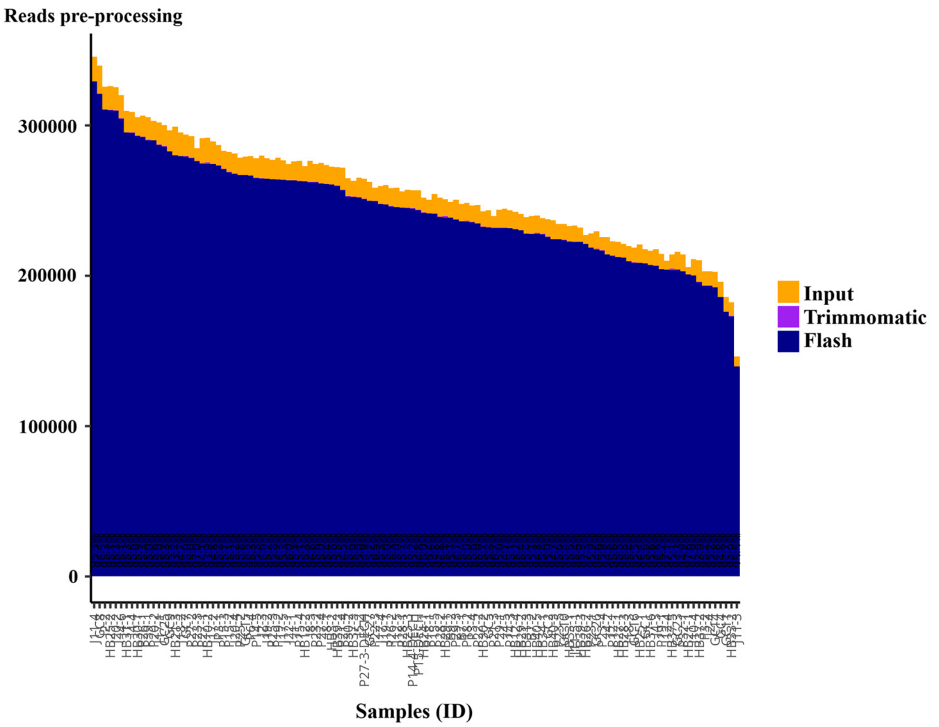

Supplementary Figure S1. Read counts per sample across the preprocessing workflow (Input, Trimmomatic, and FLASH)

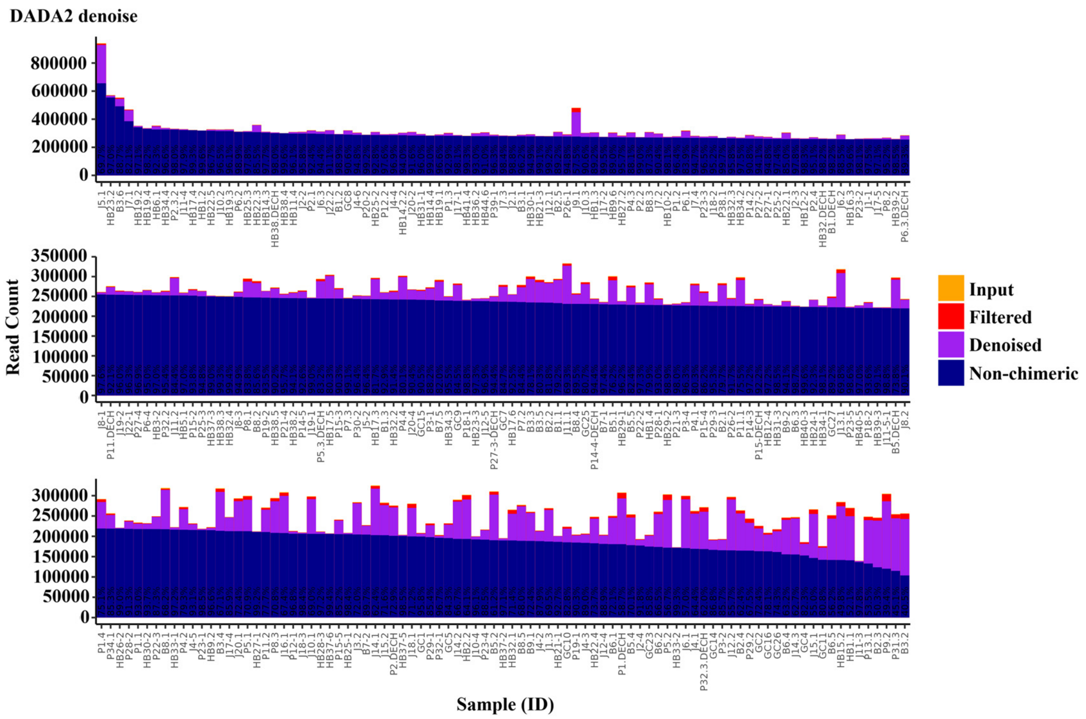

Supplementary Figure S2. Read counts per sample across the DADA2 denoising workflow (Input, Filtered, Denoised, and Non-chimeric)

2. Allogeneic Hematopoietic Stem Cell Transplantation and Control Group Data

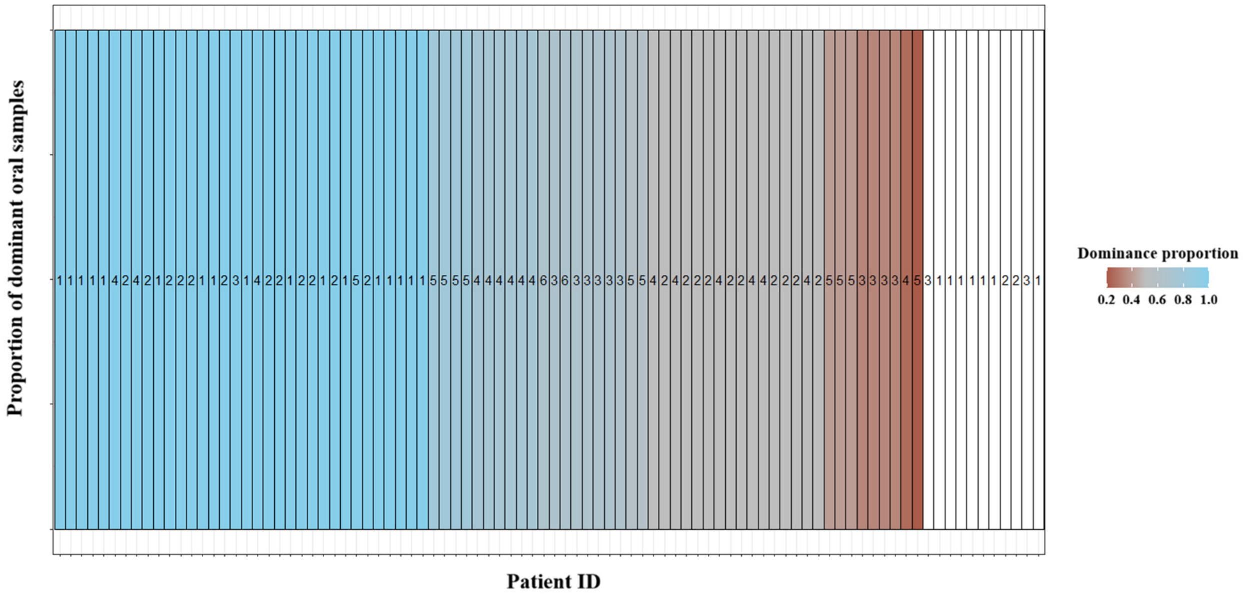

Supplementary Figure S3. Patient-level distribution of oral dominance events during allo-HSCT

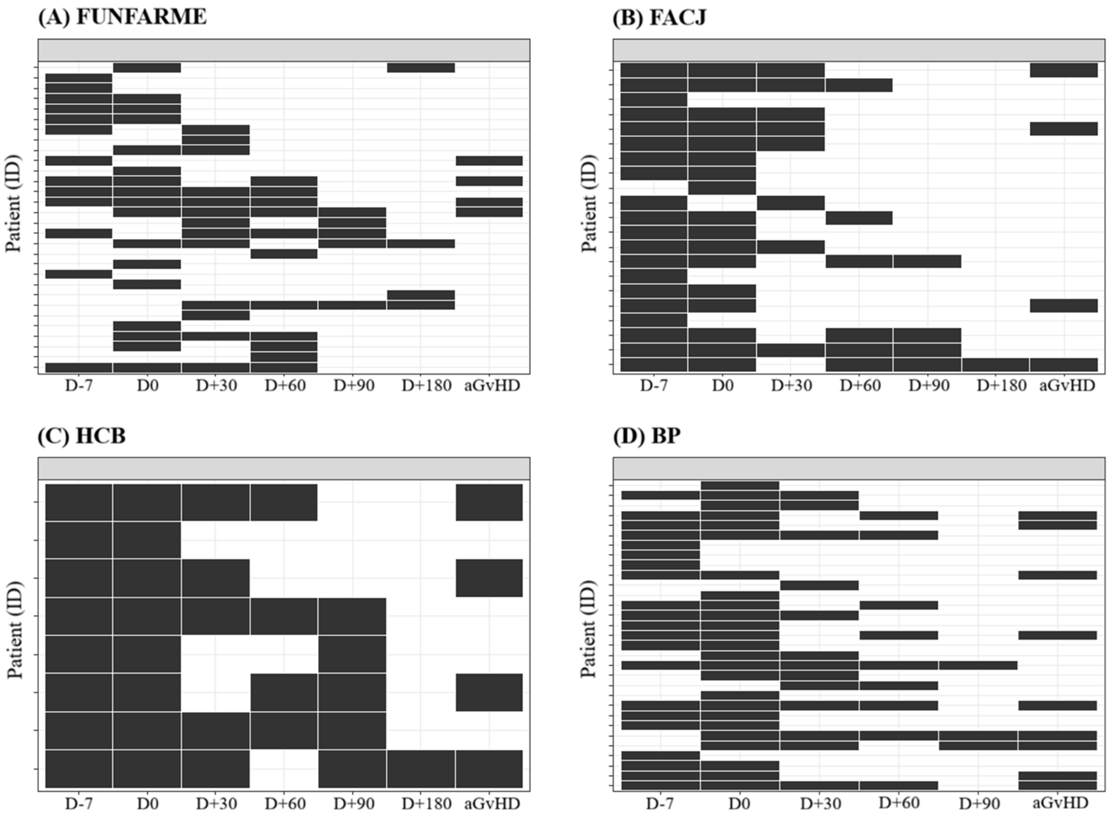

Supplementary Figure S4. Patient-level distribution of oral samples during allo-HSCT course. (A) FUNFARME - Foundation of the Regional Medical School of São José do Rio Preto; **(B)** FACJ - Amaral Carvalho Foundation; **(C)** HCB - Pio XII Foundation, Barretos Cancer Hospital; **(D)** BP - Beneficencia Portuguesa Hospital of São Paulo.

Supplementary Table S1. Distribution of oral samples across different time points and by medical center

Time point	FUNFARME*	FACJ*	HCB*	BP*
D-7	13	20	8	21
D0	17	17	8	25
D+30	13	8	5	13
D+60	11	6	4	9
D+90	5	4	5	3
D+180	4	1	1	0
aGvHD	4	4	4	9

*FUNFARME: Foundation of the Regional Medical School of Sao Jose do Rio Preto; FACJ: Amaral Carvalho Foundation; HCB: Pio XII Foundation - Barretos Cancer Hospital; BP: Beneficencia Portuguesa Hospital/São Paulo. aGvHD = acute Graft-versus-Host Disease; D = day.

Supplementary Table S2. Demographic characteristics of the control group

Variable	*n*
**Sex**	
Male (%)	8 (50%)
Female (%)	8 (50%)
**Age (years)**	
Mean (SD)	29 (8.71)
Median	26.5
Range	22-57
**Body mass index**	
Mean (SD)	23.75 (3.4)
Median	24.42
Range	18.46-28.4
**Smoking status**	
Never Smokers	16 (100%)
Current Smokers	0 (0%)
Former Smokers	0 (0%)
**Health status and eligibility criteria**	***n* (%)**
**Underlying disease**	
No	16 (100%)
Yes	0 (0%)
**Comorbidities**	
No	16 (100%)
Yes	0 (0%)
**Medication, antibiotic, probiotic, or prebiotic use**	
No	16 (100%)
Yes	0 (0%)

Supplementary Table S2. Demographic characteristics of the control group

Variable	*n*
**Current oral discomfort or pain**	
No	16 (100%)
Yes	0 (0%)
**Medical or dental visits due to oral discomfort or pain within the previous six months**	
No	16 (100%)
Yes	0 (0%)

SD = Standard deviation.

Supplementary Table S3. Association between oral *Streptococcus* dominance and blood culture results

Variable	No (N = 45)	Yes (N = 45)	*p*-value
**Positive Blood Culture_*Streptococcus***			**0.4**
**No**	44 (97.8%)	41 (91.1%)	
**Yes**	1 (2.2%)	4 (8.9%)	
**Positive Blood Culture_Other genera**			**0.5**
**No**	32 (71%)	29 (64%)	
**Yes**	13 (29%)	16 (36%)	

N = number of patients

Table S4. Weighted Unifrac pairwise post hoc comparisons

Comparison	F-exp	*p*-value	*p*-value adj.
CTRL	3.0499	0.000999	0.036
CTRL–D−7	2.8604	0.000999	0.036
CTRL–D0	4.6570	0.000999	0.036
CTRL–D+30	3.4860	0.000999	0.036
CTRL–D+60	4.0070	0.000999	0.036
CTRL–D+90	4.0976	0.000999	0.036
CTRL–D+180	4.3866	0.000999	0.036
CTRL–aGvHD	3.0001	0.000999	0.036
D−7	0.2339	0.028971	1.000
D−7–D0	0.7890	0.010989	0.396
D−7–D+30	0.7209	0.016983	0.611
D−7–D+60	0.5246	0.048951	1.000
D−7–D+90	0.4148	0.111888	1.000
D−7–D+180	0.4849	0.080919	1.000
D−7–aGvHD	0.3723	0.124875	1.000
D0	0.2554	0.014985	0.539

Table S4. Weighted Unifrac pairwise post hoc comparisons

Comparison	F-exp	*p*-value	*p*-value adj.
D0–D+30	0.7198	0.015984	0.575
D0–D+60	0.0902	0.835165	1.000
D0–D+90	0.1159	0.721279	1.000
D0–D+180	0.1752	0.505495	1.000
D0–aGvHD	0.2660	0.277722	1.000
D+30	0.3254	0.012987	0.468
D+30–D+60	0.5324	0.049950	1.000
D+30–D+90	0.6251	0.036963	1.000
D+30–D+180	0.7676	0.011988	0.432
D+30–aGvHD	0.3073	0.199800	1.000
D+60	0.1142	0.320679	1.000
D+60–D+90	0.0598	0.929071	1.000
D+60–D+180	0.1704	0.511489	1.000
D+60–aGvHD	0.1256	0.662338	1.000
D+90	0.1215	0.330669	1.000
D+90–D+180	0.0551	0.946054	1.000
D+90–aGvHD	0.1871	0.485514	1.000
D+180	0.2229	0.105894	1.000
D+180–aGvHD	0.3482	0.161838	1.000
aGvHD	0.0345	0.901099	1.000
**Total**	**5.5292**	**0.000999**	**0.000999**

D = day; CTRL = control group; aGvHD = acute Graft-versus-Host Disease

Table S5. Unweighted Unifrac pairwise post hoc comparisons

Comparison	F-exp	*p*-value	*p*-value adj.
CTRL	1.8227	0.000999	0.036
CTRL–D−7	1.3311	0.003996	0.144
CTRL–D0	2.5006	0.000999	0.036
CTRL–D+30	3.7940	0.000999	0.036
CTRL–D+60	2.6405	0.000999	0.036
CTRL–D+90	2.2239	0.000999	0.036
CTRL–D+180	1.7004	0.000999	0.036
CTRL–aGvHD	1.5485	0.000999	0.036
D−7	0.0926	0.321678	1.000
D−7–D0	0.3680	0.174825	1.000
D−7–D+30	1.0463	0.004995	0.180
D−7–D+60	0.3758	0.132867	1.000
D−7–D+90	0.3551	0.172827	1.000

Table S5. Unweighted Unifrac pairwise post hoc comparisons

Comparison	F-exp	*p*-value	*p*-value adj.
D−7–D+180	0.0990	0.767233	1.000
D−7–aGvHD	0.1044	0.739261	1.000
D0	0.1586	0.109890	1.000
D0–D+30	1.1789	0.002997	0.108
D0–D+60	0.1303	0.626374	1.000
D0–D+90	0.0953	0.784216	1.000
D0–D+180	0.2017	0.385614	1.000
D0–aGvHD	0.2898	0.242757	1.000
D+30	0.7402	0.000999	0.036
D+30–D+60	0.7636	0.015984	0.575
D+30–D+90	1.4635	0.000999	0.036
D+30–D+180	0.8847	0.005994	0.216
D+30–aGvHD	0.8641	0.006993	0.252
D+60	0.1225	0.248751	1.000
D+60–D+90	0.1989	0.407592	1.000
D+60–D+180	0.2007	0.387612	1.000
D+60–aGvHD	0.2579	0.260739	1.000
D+90	0.2058	0.127872	1.000
D+90–D+180	0.2047	0.405594	1.000
D+90–aGvHD	0.3080	0.215784	1.000
D+180	0.0352	0.945055	1.000
D+180–aGvHD	0.0656	0.953047	1.000
aGvHD	0.0560	0.733267	1.000
**Total**	**4.0612**	**0.000999**	**0.000999**

D = day; CTRL = control group; aGvHD = acute Graft-versus-Host Disease

3. Oral Domination in allo-HSCT recipients and healthy individuals

Supplementary Table S6. Distribution of dominant bacterial genera in allo-HSCT patients and healthy controls

Genus	*n* (allo-HSCT)	*N* (allo-HSCT)	*n* (control group)
Streptococcus	68	45	0
Veillonella	31	24	0
Rothia	15	12	0
Prevotella	11	10	0
Haemophilus	9	9	1
Actinomyces	7	5	0
Staphylococcus	5	4	0
Neisseria	4	4	0

Supplementary Table S6. Distribution of dominant bacterial genera in allo-HSCT patients and healthy controls

Genus	*n* (allo-HSCT)	*N* (allo-HSCT)	*n* (control group)
Arthrospira	2	1	0
Acinetobacter	2	1	0
Enterobacter	2	1	0
Klebsiella	2	2	0
Lactobacillus	2	2	0
Lautropia	2	2	0
Alloprevotella	1	1	1
Bacillus	1	1	0
Burkholderia	1	1	0
Cellulosimicrobium	1	1	0
Corynebacterium	1	1	0
Gemella	1	1	0
Jeotgalicoccus	1	1	0
Limosilactobacillus	1	1	0
Peptidiphaga	1	1	0
Pseudomonas	1	1	0
Schaalia	1	1	0
Segatella	1	1	0
Stenotrophomonas	1	1	0

N = number of patients; *n* = number of samples; allo-HSCT = allogeneic hematopoietic stem cell transplantation.

Supplementary Table S7. Distribution of dominant bacterial genera across different time points during the allo-HSCT course

Genus	*n*						
	**D-7**	**D0**	**D+30**	**D+60**	**D+90**	**D+180**	**aGvHD**
Streptococcus	18	14	10	10	6	4	6
Veillonella	1	13	7	4	4	0	2
Rothia	2	6	3	2	0	1	1
Prevotella	3	3	3	2	0	0	0
Haemophilus	5	0	2	0	1	0	1
Actinomyces	1	0	1	3	1	0	1
Staphylococcus	0	0	2	0	1	0	2
Neisseria	1	0	1	1	0	0	1
Arthrospira	0	1	1	0	0	0	1
Acinetobacter	1	0	0	0	1	0	0
Enterobacter	1	0	0	0	1	0	0
Klebsiella	0	0	1	1	0	0	0

Supplementary Table S7. Distribution of dominant bacterial genera across different time points during the allo-HSCT course

Genus	*n*						
	**D-7**	**D0**	**D+30**	**D+60**	**D+90**	**D+180**	**aGvHD**
Lactobacillus	0	1	0	0	0	0	1
Lautropia	0	1	1	0	0	0	0
Alloprevotella	0	0	1	0	0	0	0
Bacillus	0	1	0	0	0	0	0
Burkholderia	0	0	0	1	0	0	0
Cellulosimicrobium	0	0	0	0	0	0	1
Corynebacterium	0	0	0	1	0	0	0
Gemella	1	0	0	0	0	0	0
Jeotgalicoccus	0	0	0	1	0	0	0
Limosilactobacillus	0	1	0	0	0	0	0
Peptidiphaga	0	1	0	0	0	0	0
Pseudomonas	0	0	1	0	0	0	0
Schaalia	0	1	0	0	0	0	0
Segatella	1	0	0	0	0	0	0
Stenotrophomonas	0	0	1	0	0	0	0

n = number of samples; allo-HSCT = allogeneic hematopoietic stem cell transplantation; aGvHD = acute Graft-versus-Host Disease; D = day.

4. Associations between oral dominance by specific genus and clinical outcomes

Supplementary Table S8. Cumulative incidence estimates of clinical outcomes according to oral dominance status during follow-up

<table>
<tr><th>Outcome</th><th>Oral dominance</th><th>Time (days)</th><th>N at risk</th><th>Cumulative incidence % (95% CI)</th><th>Gray's p-value</th></tr>
<tr><td rowspan="8">Death within D+180</td><td rowspan="4">No</td><td>50</td><td>8</td><td>22.2 (2.8–53.1)</td><td rowspan="8">0.06</td></tr>
<tr><td>100</td><td>8</td><td>22.2 (2.8–53.1)</td></tr>
<tr><td>150</td><td>7</td><td>33.3 (6.7–64.0)</td></tr>
<tr><td>200</td><td>5</td><td>44.4 (11.7–73.6)</td></tr>
<tr><td rowspan="4">Yes</td><td>50</td><td>57</td><td>2.6 (0.5–8.1)</td></tr>
<tr><td>100</td><td>47</td><td>7.0 (2.6–14.5)</td></tr>
<tr><td>150</td><td>40</td><td>13.1 (6.4–22.4)</td></tr>
<tr><td>200</td><td>29</td><td>18.3 (9.9–28.7)</td></tr>
<tr><td rowspan="5">Moderate-to-severe aGvHD</td><td rowspan="4">No</td><td>50</td><td>8</td><td>0.0 (NE)</td><td rowspan="5">0.15</td></tr>
<tr><td>100</td><td>8</td><td>0.0 (NE)</td></tr>
<tr><td>150</td><td>7</td><td>0.0 (NE)</td></tr>
<tr><td>200</td><td>5</td><td>0.0 (NE)</td></tr>
<tr><td>Yes</td><td>50</td><td>57</td><td>16.3 (8.9–25.7)</td></tr>
</table>

Supplementary Table S8. Cumulative incidence estimates of clinical outcomes according to oral dominance status during follow-up

Outcome	Oral dominance	Time (days)	N at risk	Cumulative incidence % (95% CI)	Gray's *p*-value
		100	47	19.3 (11.1–29.1)	
		150	40	19.3 (11.1–29.1)	
		200	29	19.3 (11.1–29.1)	
**Disease relapse**	No	50	8	0.0 (NE)	**0.31**
		100	8	0.0 (NE)	
		150	7	0.0 (NE)	
		200	5	0.0 (NE)	
	Yes	50	57	1.3 (0.1–6.4)	
		100	47	4.3 (1.1–11.0)	
		150	40	5.8 (1.9–13.2)	
		200	29	11.0 (4.7–20.2)	

NE = not estimable (no events occurred before the specified time point); N = number of patients; aGvHD = acute Graft-versus-Host Disease; D = day.

Supplementary Table S9. Cumulative incidence estimates of clinical outcomes according to *Streptococcus* oral dominance status during follow-up

Outcome	Oral dominance	Time (days)	N at risk	Cumulative incidence % (95% CI)	Gray's *p*-value
**Death within D+180**	No	50	34	7.2 (1.8–17.8)	**0.40**
		100	29	7.2 (1.8–17.8)	
		150	26	10.2 (3.1–22.2)	
		200	20	17.0 (6.5–31.5)	
	Yes	50	31	2.2 (0.2–10.3)	
		100	26	9.6 (3.0–21.0)	
		150	21	19.5 (9.0–33.0)	
		200	14	24.8 (12.6–39.1)	
**Moderate-to-severe aGvHD**	No	50	34	7.5 (1.9–18.4)	**0.029**
		100	29	7.5 (1.9–18.4)	
		150	26	7.5 (1.9–18.4)	
		200	20	7.5 (1.9–18.4)	
	Yes	50	31	21.3 (10.4–34.7)	
		100	26	26.2 (13.9–40.2)	
		150	21	26.2 (13.9–40.2)	
		200	14	26.2 (13.9–40.2)	
**Disease relapse**	No	50	34	2.4 (0.2–11.2)	**0.52**
		100	29	8.0 (2.0–19.5)	
		150	26	10.9 (3.4–23.6)	

Supplementary Table S9. Cumulative incidence estimates of clinical outcomes according to *Streptococcus* oral dominance status during follow-up

Outcome	Oral dominance	Time (days)	N at risk	Cumulative incidence % (95% CI)	Gray's *p*-value
		200	20	10.9 (3.4–23.6)	
	Yes	50	31	0.0 (NE)	
		100	26	0.0 (NE)	
		150	21	0.0 (NE)	
		200	14	8.3 (2.0–20.5)	

NE = not estimable (no events occurred before the specified time point); N = number of patients; aGvHD = acute Graft-versus-Host Disease; D = day.

Supplementary Table S10. Cumulative incidence estimates of clinical outcomes according to *Veillonella* oral dominance status during follow-up

Outcome	Oral dominance	Time (days)	N at risk	Cumulative incidence % (95% CI)	Gray's *p*-value
**Death within D+180**	No	50	48	4.8 (1.2–12.2)	**0.92**
		100	42	8.2 (3.0–16.9)	
		150	35	17.2 (8.7–28.0)	
		200	24	21.0 (11.5–32.5)	
	Yes	50	17	4.2 (0.3–18.0)	
		100	13	9.6 (1.5–27.1)	
		150	12	9.6 (1.5–27.1)	
		200	10	21.0 (6.0–42.0)	
**Moderate-to-severe aGvHD**	No	50	48	16.4 (8.4–26.8)	**0.30**
		100	42	19.8 (10.9–30.8)	
		150	35	19.8 (10.9–30.8)	
		200	24	19.8 (10.9–30.8)	
	Yes	50	17	9.2 (1.5–25.8)	
		100	13	9.2 (1.5–25.8)	
		150	12	9.2 (1.5–25.8)	
		200	10	9.2 (1.5–25.8)	
**Disease relapse**	No	50	48	1.6 (0.1–7.7)	**0.26**
		100	42	1.6 (0.1–7.7)	
		150	35	1.6 (0.1–7.7)	
		200	24	7.8 (2.4–17.4)	
	Yes	50	17	0.0 (NE)	
		100	13	10.8 (1.7–29.8)	
		150	12	16.2 (3.8–36.5)	
		200	10	16.2 (3.8–36.5)	

NE = not estimable (no events occurred before the specified time point); N = number of patients; aGvHD = acute Graft-versus-Host Disease; D = day.

Supplementary Table S11. Cumulative incidence estimates of clinical outcomes according to *Rothia* oral dominance status during follow-up

Outcome	Oral dominance	Time (days)	N at risk	Cumulative incidence % (95% CI)	Gray's *p*-value
**Death within D+180**	No	50	54	5.4 (1.7–12.2)	**0.69**
		100	45	8.5 (3.4–16.5)	
		150	37	16.6 (8.7–26.6)	
		200	29	21.6 (12.4–32.5)	
	Yes	50	11	0.0 (NE)	
		100	10	8.3 (0.4–32.4)	
		150	10	8.3 (0.4–32.4)	
		200	5	21.7 (2.3–54.0)	
**Moderate-to-severe aGvHD**	No	50	54	15.6 (8.2–25.0)	**0.39**
		100	45	18.7 (10.5–28.7)	
		150	37	18.7 (10.5–28.7)	
		200	29	18.7 (10.5–28.7)	
	Yes	50	11	8.3 (0.4–32.3)	
		100	10	8.3 (0.4–32.3)	
		150	10	8.3 (0.4–32.3)	
		200	5	8.3 (0.4–32.3)	
**Disease relapse**	No	50	54	1.4 (0.1–6.6)	**0.046**
		100	45	4.5 (1.2–11.5)	
		150	37	6.1 (1.9–13.8)	
		200	29	6.1 (1.9–13.8)	
	Yes	50	11	0.0 (NE)	
		100	10	0.0 (NE)	
		150	10	0.0 (NE)	
		200	5	30.0 (5.2–61.2)	

NE = not estimable (no events occurred before the specified time point); N = number of patients; aGvHD = acute Graft-versus-Host Disease; D = day.
